# The Association between Ovarian Cancer and the Incidence of Newly Developed Dry Eye Disease: A Nationwide Population-Based Study

**DOI:** 10.3390/life14040530

**Published:** 2024-04-21

**Authors:** Chia-Yi Lee, Shun-Fa Yang, Yu-Ling Chang, Jing-Yang Huang, Chao-Kai Chang

**Affiliations:** 1 Institute of Medicine, Chung Shan Medical University, Taichung 402, Taiwan; 2 Nobel Eye Institute, Taipei 115, Taiwan; 3 Department of Ophthalmology, Jen-Ai Hospital Dali Branch, Taichung 412, Taiwan; 4 Department of Medical Research, Chung Shan Medical University Hospital, Taichung 402, Taiwan; 5 Department of Medical Education, Cathay General Hospital, Taipei 106, Taiwan; 6 Department of Optometry, Da-Yeh University, Chunghua 515, Taiwan

**Keywords:** dry eye disease, ovarian cancer, oxidative stress, age, epidemiology

## Abstract

We aim to investigate the potential correlation between the presence of ovarian cancer and the development of dry eye disease (DED) via the usage of the Longitudinal Health Insurance Database (LHID) of Taiwan. A retrospective cohort study was executed, and patients with ovarian cancer were selected according to the diagnostic and procedure codes. One ovarian cancer patient was matched to four non-ovarian cancer participants which served as control group, and a total of 4992 and 19,968 patients constructed the ovarian cancer and control groups, respectively. The primary outcome in the current study is the development of DED according to the diagnostic and procedure codes. Cox proportional hazard regression was utilized to produce the adjusted hazard ratio (aHR) and related 95% confidence interval (CI) of DED between the two groups. There were 542 and 2502 DED events observed in the ovarian cancer group and the control group, respectively. The ovarian cancer group illustrated a significantly higher incidence of DED development than the control group after the adjustment of several confounders (aHR: 1.10, 95% CI: 1.01–1.21, *p* = 0.040). In the subgroup analysis stratified by age, ovarian cancer patients aged older than 60 years showed a higher incidence of DED compared to the non-ovarian cancer population (aHR: 1.19, 95% CI: 1.08–1.28, *p* = 0.011). In addition, ovarian cancer patients with a disease duration longer than five years also showed higher incidence of DED formation than the non-ovarian cancer population (aHR: 1.13, 95% CI: 1.04–1.22, *p* = 0.027). In conclusion, the presence of ovarian cancer is associated with higher incidence of subsequent DED, especially in those older than 60 years and with a disease interval of more than five years.

## 1. Introduction

Ovarian cancer is a common gynecological cancer in the world, which is the fifth leading etiology of cancer-related mortality among women in the American population [1,2]. The risk factors of the ovarian cancer involved delayed childbearing, usage of hormone replacement therapy, early menarche, oral contraceptive usage, family history of ovarian cancer, obesity, and pre-existing endometriosis [1,3,4]. The treatment options for ovarian cancer include the surgical removal and chemotherapy, and bevacizumab has gained attention in the recent years [5,6]. Despite the advance of therapy, the prognosis of ovarian cancer is still poor, with a five-year survival rate lower than 50 percent [7].

There are several pathophysiologies regarding the development of ovarian cancer in pervious research [8,9,10]. The steroid hormone effects of progesterone and estrogen in ovarian environment correlate to ovarian cancer formation [11], and the gene mutation, such as BRCA1 and BRCA2 genes, elevated the risk of high-grade serous ovarian carcinoma [12]. In addition, the oxidative stress is another crucial mechanism for the development of ovarian cancer [13,14]. Higher reactive oxygen species, including inducible nitric oxide synthase and superoxide dismutase, was observed in individuals with ovarian cancer [15]. In addition, the antioxidant enzyme NAD(P)H:quinone oxidoreductase 1 could regulate the response to chemotherapy in ovarian cancer [16], and the antioxidant-related gene NRF2 has been shown to be an indicator for the prognosis of ovarian cancer [17].

Several conditions that associated with ovarian cancer were reported [18,19]. The type-2 diabetes mellitus (T2DM) revealed strong association to the poor prognosis of ovarian cancer in previous analysis [20]. Other metabolic syndromes like hyperlipidemia also correlate to increasing risk of ovarian cancer development [21]. Ovarian cancer tends to occur in patients with higher body mass index and obesity [4]. In addition, dementia is a disease related to oxidative stress [22], occurring in 2.1 percent of ovarian cancer individuals [23].

Dry eye disease (DED) is an ocular surface disorder that relates to meibomian gland dysfunction (MGD) and loss of tear film homeostasis [24,25,26]. Previous publications illustrated a positive relationship between DED occurrence and several diseases such as Sjogren syndrome, T2DM, and the rheumatic arthritis [27]. For further mechanisms, DED is an ocular surface disease that characterized with loss of tear film homeostasis and the increment of both inflammation and oxidative stress [28,29,30]. In a preceding article, both the expressions of interleukin-6 and the reactive oxygen species are significantly higher in the individuals with DED [25]. In addition, certain systemic morbidities, including Sjogren syndrome and systemic lupus erythematous, which serve as the predisposing factors for DED formation, are associated with inflammatory response [27,31]. Regarding DED and ovarian cancer, there is limited research that discusses the relationship between them. Because higher oxidative stress presents in both ovarian cancer and DED [13,32], there may be a correlation between these two diseases which need further validation.

The purpose of the current study is to evaluate the potential correlation between the presence of ovarian cancer and the development of subsequent DED events. 

## 2. Materials and Methods

### 2.1. Data Resource

Females in the National Health Insurance Research Database (NHIRD) system were used for the performance of our analyses. The Taiwan NHIRD records the medical consultation documents of the 23 million people living in Taiwan from 1 January 2000 to 31 December 2020. The available medical information in Taiwan NHIRD are presented as follows: the International Classification of Diseases-Ninth Revision (ICD-9) of diagnostic code, the International Classification of Diseases-Tenth Revision (ICD-10) of diagnostic code, age, sex, occupation, income amount, urbanization intensity, education level, the image-based codes, laboratory exam-based codes, medical department-related codes, procedure/surgery codes, and finally, the international ATC codes for medicine. Only the image exam, laboratory exam, procedure/surgery, and medicine offered by the Taiwan national health insurance system are available in the NHIRD. The Taiwan Longitudinal Health Insurance Database (LHID) 2000 is a sub-database of Taiwan NHIRD, and was applied to the analysis in the current study. The LHID 2000 contains about two million people that were randomly drafted from the NHIRD using the automated software, and the available information in the LHID 2000 is the same as those in the NHIRD.

### 2.2. Study Population Selection

**Figure 1:**
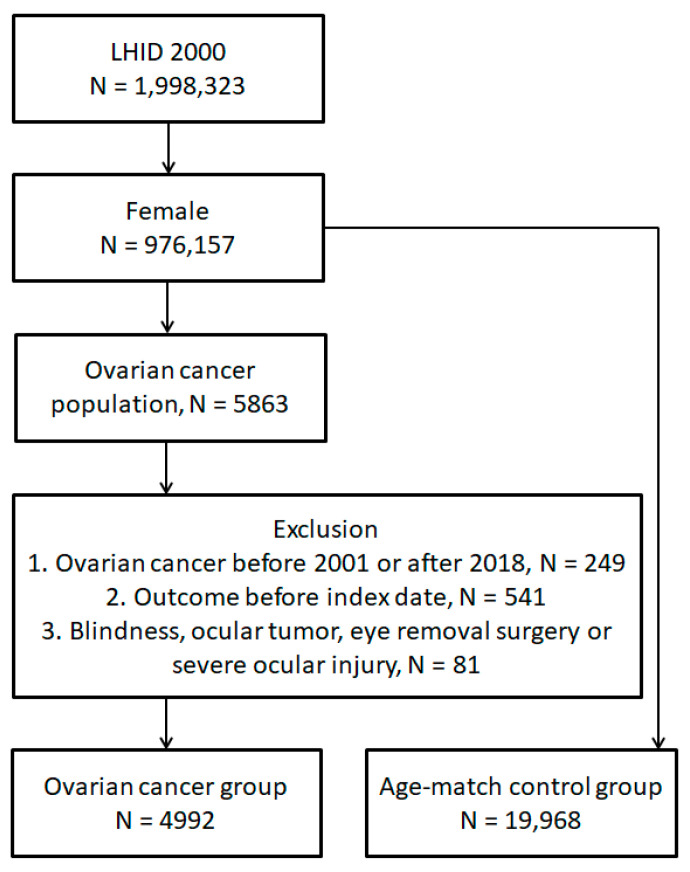
The flowchart of subject selection. N: number, LHID: Longitudinal Health Insurance Database.

A retrospective cohort study was executed. The patients in the LHID 2000 were regarded as having ovarian cancer if completed the subsequent criteria: (1) ovarian cancer diagnosis according to corresponding ICD-9 plus ICD-10 diagnostic codes; (2) the completion of a pelvic exam before the day of the ovarian cancer diagnosis, according to the procedure/surgery codes; (3) the completion of a pelvic ultrasound exam, computed tomography, or the cancer antigen 125 test before the day of the ovarian cancer diagnosis, according to the procedure/surgery codes; and (4) the ovarian cancer was diagnosed in the gynecological department. The index date was designated as the six months after the emergence of the ovarian cancer-related diagnosis. The following exclusion criteria was adopted: (1) the ovarian cancer was diagnosed before 2001 or after 2019, to standardize the timeliness of ovarian cancer; (2) the primary outcome occurred before the index date; (3) the blindness occurred before the index date, in accordance with corresponding ICD-9 and ICD-10 diagnosis codes; (4) the ophthalmic tumor occurred before the index date, in accordance with corresponding ICD-9 and ICD-10 diagnosis codes; (5) the eye removal intervention was arranged before the index date, in accordance with corresponding intervention/surgery codes; and (6) the severe ocular injury occurred before the index date, in accordance with corresponding ICD-9 and ICD-10 diagnosis codes. For comparison, one ovarian cancer patient was age-matched to four non-ovarian cancer individuals, and the latter population constituted the control group. The reason for a 1:4 match is because we wanted to include as many cases as possible to reduce the bias from low patient numbers. However, if we matched 1 ovarian cancer patient to 8 or 10 non-ovarian cancer patients, the extreme discordance of the patient number between the two groups may have contributed to statistical bias. As a result, after discussing with our statistical specialist and consult previous publications [33,34], we decide to execute the match process with 1:4. A total of 4992 and 19,968 participants were enrolled into the ovarian cancer group and the control group, respectively. The flowchart of patient selection is available as Figure 1.

### 2.3. Primary Outcome

The primary outcome of the current study is the development of DED, which is based on the following conditions: (1) a diagnosis of DED according to corresponding ICD-9 and ICD-10 diagnostic codes; (2) the arrangement of slit-lamp biomicroscope exam before the DED diagnosis according to the corresponding procedure/surgery code; (3) the arrangement of fluorescein stain test or Schirmer test before the DED diagnosis according to the corresponding procedure/surgery codes; (4) the prescription of artificial tear after the diagnosis of DED according to corresponding ATC codes; and (5) the DED diagnosis was made in the ophthalmic department. To better establish the time sequence between the ovarian cancer episode and following DED development, only the DED events found after the index date were accounted as outcome achievement. For the evaluation of primary outcome, the participants in the current study were tracked until the development of DED, discarded from the national health insurance program, or up to the deadline of NHIRD/LHID 2000, which indicate a date of 31 December 2020.

### 2.4. Covariates and Co-Morbidities

In addition to the primary outcome, several confounders were included in the analysis model to adjust the effect of these factors on DED development as possible: age, urbanization, hypertension, T2DM, ischemic heart diseases, hyperlipidemia, peripheral vascular disease, end-stage renal disease, rheumatoid arthritis, systemic lupus erythematosus, Sjogren syndrome, MGE/Blepharitis, and the receipt of cataract surgery. The presence of these covariates were based on the demographic codes, ICD-9 and ICD-10 diagnostic codes, and the procedure/surgery codes in the NHIRD/LHID 2000. In addition, only the diseases that diagnosed for more than two years were enrolled as the confounder to ensure the disease interval and associated effect on DED formation.

### 2.5. Statistical Analysis

The SAS edition 9.4 version (SAS Institute Inc., Cary, NC, USA) was adopted for all the statistical analyses in current study. Descriptive analysis were used to present the demographic data and co-morbidities of the two groups, and the normality of study population was tested and affirmed using the Shapiro–Wilk test before subsequent analysis (*p* > 0.05). The standard mean difference (SMD) was applied to compare the distribution of each parameter between the two populations, and an SMD amount > 0.1 was regarded as the significant difference. Then, the Cox proportional hazard regression analysis was executed to produce an adjusted hazard ratio (aHR) and related 95 percent confidence interval (CI) of DED development between the two populations. The effect of age, urbanization, and DED-associated co-morbidities were all adjusted in the Cox proportional hazard regression analysis. In the subgroup analyses, the ovarian cancer patients were stratified according to age (<40 years, 40–60 years, and <60 years) and duration of ovarian cancer (<2 years, 2–4 years, and >4 years). The Cox proportional hazard regression was adopted again with the adjustments of all the co-morbidities, and yielded the aHR and corresponding 95% CI. The Cox proportional hazard regression belongs to the family of survival analysis, which can present the ratio of an exposure population (the ovarian cancer group in the current study) surviving from an outcome/morbidity (which is DED in the current study). In addition to the comparison between exposure and non-exposure groups, the Cox proportional hazard regression can integrate the effect of multiple factors in the model; thus, the effect of known risk factors of DED like age, T2DM, and Sjogren syndrome can be adjusted in the analysis to elevate the accuracy of our results. When compared to other multiple regression analyses, the Cox proportional hazard regression can put the survival time (from exposure to outcome or the end of study period) in the analysis model, and the analysis can be better refined. Finally, a *p* < 0.05 was set as statistical significance in the current study, and *p* values underneath 0.001 were depicted as *p* < 0.001.

## 3. Results

The baseline characteristics are presented in Table 1. The age distributions between the two groups were not-significantly different due to the matching process (SMD = 0.002). The level of urbanization was also similar between the two groups (SMD = 0.007). Regarding the co-morbidities, the distribution of T2DM, ischemic heart disease, and blepharitis/MGD were significantly higher in the ovarian cancer group than the control group (all SMD > 0.1), while the rests of co-morbidities showed similar distribution between the two groups (all SMD < 0.1) (Table 1).

After the follow-up interval, there were 542 and 2502 episodes of DED found in the ovarian cancer group and the control group, respectively (Table 2). The ovarian cancer group demonstrated a significantly higher incidence of DED development than the control group, with the adjustment of several confounders (aHR: 1.10, 95% CI: 1.01–1.21, *p* = 0.040) (Table 2). In the subgroup analysis stratified by age, the ovarian cancer patients younger than 60 years did not show higher incidence of DED than the control group (both *p* > 0.05), but the ovarian cancer patients older than 60 years revealed a higher incidence of DED compared to the non-ovarian cancer population (aHR: 1.19, 95% CI: 1.08–1.28, *p* = 0.011). On the other hand, the ovarian cancer patients with disease duration more than five years showed higher chance for developing DED than the non-ovarian cancer population (aHR: 1.13, 95% CI: 1.04–1.22, *p* = 0.027). Still, the ovarian cancer individuals with disease duration lesser than five years showed similar rate of DED occurrence compared to the non-ovarian cancer population (both *p* > 0.05) (Table 3).

## 4. Discussion

Briefly, the current study demonstrated a higher incidence of later DED in the patients diagnosed with ovarian cancer. Moreover, the incidence of DED was even higher in the ovarian cancer patients older than 60 years. On the other hand, the patients diagnosed with ovarian cancer for more than five years presented with a significantly higher risk of developing DED than the non-ovarian cancer population.

The patients diagnosed with ovarian cancer associated with a higher incidence of subsequent DED in the current study. In a previous study, the existence of ovarian cancer showed a significant relationship to several diseases like the obesity and T2DM [4,20]. Still, no evidence for the correlation between ovarian cancer and eye disease was proposed previously. In some experimental research, oxidative stress on the ocular surface was significantly elevated in the individuals with moderate DED [25,35]. Regarding the correlation between antioxidant and DED development, a previous study demonstrated that the higher amount of antioxidant may decrease the severity of DED signs and symptoms [36]. Since both the ovarian cancer and DED share similar mechanism of elevated oxidative stress [15,25,37], the presence of ovarian cancer may indicate a higher baseline oxidative stress, and the incidence of DED may be elevated in such condition. This concept was supported by the results of the current study. To our knowledge, this may be a preliminary experience to demonstrate the positive correlation between the ovarian cancer and the following DED development. Moreover, we excluded the patients diagnosed with DED before or within 6 months after the diagnosis of ovarian cancer; thus, the time sequence between the two diseases was be established. In addition, we adjusted the effect of several predisposing factors of DED including age, T2DM, Sjogren syndrome, MGD, and cataract surgery in the Cox proportional hazard regression [38,39]. Consequently, the ovarian cancer may be an independent risk factor for the development of DED. Oxidative stress possibly served as the main mechanism for the ovarian cancer-to-DED relationship because inflammation and elevated oxidative stress were regarded as the major pathophysiologies for many eye diseases including DED [25,40]. On the other hand, ovarian cancer did not raise systemic inflammation prominently, but could contribute to the increasing serum oxidative stress [15], which can circulate to the whole body involve the orbital and ocular region. As a consequence, elevated oxidative stress could be the reason for the increasing DED incidence in the ovarian cancer population. In addition to oxidative stress, serum sex hormones level like estrogen are crucial for the onset of DED during in vitro fertilization [41], and both the elevation of estrogen and progesterone are associated with the development of DED, despite a few papers demonstrating contrary results [42]. Since ovarian cancer featured with increased estrogen and progesterone [11], this may be another mechanism for DED development in individuals with ovarian cancer. 

On the other hand, the percentage of DED was numerically higher in the control group compared to the ovarian cancer group, which is theoretically contrary to the results of Cox proportional hazard regression. Still, the follow-up person-month per patient was also higher in the control group than that in the ovarian cancer group. Consequently, it may be reasonable that the percentage of DED is slightly higher in the population with longer follow up period. Because the Cox proportional hazard regression can consider the effect of exposure-to-event time on the incidence of outcome, the incidence of DED became significantly higher in the ovarian cancer group after the adjustment. The longer follow-up person-per-month in the control group may result from the fact that the population with ovarian cancer experiences a higher mortality rate than the normal population, and the mean age of ovarian cancer patients was significantly shorter in the previous study.

In the subgroup analysis, ovarian cancer patients aged older than 60 years showed a higher incidence of DED compared to the non-ovarian-cancer individuals, and the aHR of DED in the ovarian cancer subgroups toward non-ovarian-cancer subgroups numerically decreased with younger age. Age is a known risk factor for DED, in which the prevalence of DED elevates every five years in the population older than 50 years [43]. Also, the tear volume was significantly lower in patients older than 40 years [44]. On the other hand, ovarian cancer tends to occur in the female population older than 50 years old, which shows an incidence rate higher than 25 per 100,000 women [3]. In addition to the role in the development of the two diseases, age is also related to increased oxidative stress [44]. Due to the metabolism, the reactive oxygen species expression was significantly increased in the elderly population [45]. Consequently, old age may lead to higher rate of ovarian cancer, and both of these factors are associated with higher oxidative stress; thus, DED occurred much easier in this situation. Regarding the influence of disease duration, the ovarian cancer patient with a disease period longer than five years showed a higher chance for developing DED. There was sparse research which reported this phenomenon. The moderate oxidative stress level can promote the proliferation of ovarian cancer [15]; thus, we speculate that the persistent oxidative stress that accompanies prolonged ovarian cancer course could cause more damage to the human body, including the ocular surface. In addition, ovarian cancer progresses over time, and the advanced stage of ovarian cancer may contribute to a higher level of oxidative stress. Nevertheless, further study is needed to confirm this hypothesis.

Concerning the epidemiology, the ovarian cancer is the second most frequent gynecological cancer worldwide, just after breast cancer [7]. There were more than 200,000 patients diagnosed with ovarian cancer in 2018 [7], and more than 100,000 women die of ovarian cancer annually throughout the world [1]. North America, Europe, and Oceania showed the highest mortality rate from ovarian cancer [46]. DED, similarly to ovarian cancer, affects numerous people, with an annual incidence of more than five percent in men and women older than 40 years [27,47]. In specific situations, like the population using a visual display terminal frequently, the incidence of DED can reach a rate as high as 60 percent [48]. Advanced DED can not only damage the ocular surface, but can also contribute to significant visual impairment and depressive conditions [27]. Because both DED and ovarian cancer affects a huge number of people and would contribute to tremendous cost and disability, any correlation between them may be demonstrated to better prevent the development of these two prevalent diseases.

There are several major limitations in the current study. Firstly, both the NHIRD and LHID 2000 are claimed databases which only preserve the codes for diagnosis, examination, procedure, and medication prescription. As a consequence, much crucial information, including the severity of co-morbidities, the site of ovarian cancer, the laterality of ovarian cancer, the detailed pathological report of ovarian cancer, the results of image and laboratory exam for ovarian cancer, the TNM stage for ovarian cancer, the treatment response of ovarian cancer, the detail of surgery for ovarian cancer if arranged, the recurrence of ovarian cancer, the type of DED, the external eye image of DED, the results of DED-related examination, the severity of DED, the grade of MGD in DED population, the treatment response of DED, and the subjective symptom of DED, cannot be accessed or cannot be accessed with enough accuracy. Similarly, we cannot obtain the serum sex hormones level in the Taiwan NHIRD because it only contained claimed data. In addition, the contact lens wear and the receipt of refractive surgery are prominent risk factors for the development of DED [38], but the two covariates cannot be evaluated via the NHIRD and LHID 2000 because both managements are self-paid in Taiwan, which will not be recorded in the national health insurance system. In addition, nearly all the participants in the current study are Taiwanese, and thus the external validity of our results is limited. Finally, the ovarian cancer group showed a higher ratio of T2DM, ischemic heart disease, and MGD, which leads to a high heterogeneity between the two groups concerning the evaluation of DED risk. However, we applied the Cox proportional hazard regression to adjust the effect of these parameters. As a result, the influence of heterogeneity on the accuracy of our analysis might not be prominent.

## 5. Conclusions

In conclusion, the existence of ovarian cancer is associated with higher incidence of subsequent DED after the adjustments of multiple risk factors. Furthermore, the risk of DED in the ovarian cancer population increase with the aging and prolonged disease course. Consequently, the routine ophthalmic examination for the ovarian cancer patients older than 60 years or diagnosed for more than five years could be recommended. A further large-scale prospective study to reveal whether the presence of ovarian cancer would influence the therapeutic outcome of DED is necessary.

## Figures and Tables

**Table 1:** Baseline characteristic between the study groups.

Characteristic	Control Group (N = 19,968)	Ovarian Cancer Group (N = 4992)	SMD
Age at index date			0.002
<40	5965 (29.87%)	1490 (29.85%)	
40–49	5127 (25.68%)	1267 (25.38%)	
50–59	4099 (20.53%)	1038 (20.79%)	
60–69	2369 (11.86%)	589 (11.80%)	
70–79	1567 (7.85%)	398 (7.97%)	
≥80	841 (4.21%)	210 (4.21%)	
Urbanization			0.007
1 (high)	6637 (33.24%)	1714 (34.33%)	
2	5911 (29.60%)	1524 (30.53%)	
3	3103 (15.54%)	775 (15.52%)	
4	2584 (12.94%)	604 (12.10%)	
5	365 (1.83%)	85 (1.70%)	
6	793 (3.97%)	165 (3.31%)	
7 (low)	575 (2.88%)	125 (2.50%)	
Co-morbidity			
Hypertension	3237 (16.21%)	947 (18.97%)	0.045
T2DM	1523 (7.63%)	468 (9.38%)	0.398 *
Ischemic heart diseases	755 (3.78%)	248 (4.97%)	0.117 *
Hyperlipidemia	1680 (8.41%)	448 (8.97%)	0.017
Peripheral vascular disease	92 (0.46%)	24 (0.48%)	0.005
End-stage renal disease	78 (0.39%)	42 (0.84%)	0.076
Rheumatoid arthritis	121 (0.61%)	37 (0.74%)	0.031
Systemic lupus erythematosus	23 (0.12%)	11 (0.22%)	0.009
Sjogren Syndrome	184 (0.92%)	49 (0.98%)	0.010
Blepharitis/MGD	976 (4.88%)	296 (5.92%)	0.249 *
Cataract surgery	250 (1.25%)	61 (1.22%)	0.003

T2DM: type 2 diabetes mellitus, MGD: meibomian gland dysfunction, N: number, SMD: standard mean difference. * denotes significant difference between groups.

**Table 2:** Risk of dry eye disease between the two groups.

Dry Eye Event	Control Group (N = 19,968)	Ovarian Cancer Group (N = 4992)	*p* Value
Follow-up person-month	1,875,677	375,612	
Cases	2502	542	
Crude HR (95% CI)	Reference	1.08 (0.98–1.18)	
aHR (95% CI)	Reference	1.10 (1.01–1.21)	0.040 *

aHR: adjusted hazard ratio, adjusted for demographics and co-morbidities, CI: confidence interval, N: number. * denotes significant difference between groups.

**Table 3:** Subgroup analysis stratified by age and duration of ovarian cancer.

Subgroup	aHR ^#^	95% CI	*p* Value
Age			
<40 years	0.92	0.76–1.01	0.264
41–60 years	1.05	0.97–1.11	0.355
>60 years	1.19	1.08–1.28	0.011 *
Duration of ovarian cancer			
<2 years	1.04	0.92–1.14	0.319
2–5 years	1.00	0.94–1.13	0.186
>5 years	1.13	1.04–1.22	0.027 *

aHR: adjusted hazard ratio, adjusted for demographics and co-morbidities, CI: confidence interval. * Denotes significant difference between groups. ^#^ Risk of dry eye in ovarian cancer population compared to non-ovarian cancer population.

## Data Availability

The original data used in the current study are not available due to the policy of Taiwan National Health Insurance Administration.
